# Extraction of Needles from the Flesh

**Published:** 1846-03

**Authors:** 


*Extraction of Needles from the Flesh.*—The following statement is taken from the Sheffield Mercury, and certainly, from what appears in the imperfect state of the report, presents some curious circumstances for consideration. The degree of credit to be attached to it we leave to our readers, premising only that one very remarkable circumstance, and seeming to indicate a more recent introduction of the needles than the correspondent of the paper seems to imply, is, that their polish was not at all affected. It most generally happens in such cases that the needles are corroded to a greater or less extent: "A striking illustration of one of this class of cases has occurred in Sheffield in the course of this week. The subject in this instance is a servant in the family of Mrs. Heppenstall, of Upperthorpe, and about 21 or 22 years of age. It seems the young woman was recently under the care of a medical man whose treatment produced salivation, subsequent to which she fancied that needles were slowly progressing from her left shoulder to the arm, at some depth in the muscular substance. On Monday the pain became excruciating, and presently one, and soon afterwards three small needles were extracted from the fleshy part of the arm; and on the following morning two more, with part of a third! They made their appearance partly above and partly below the elbow, and seem perfectly bright and uncorroded. Mr. John Heppenstall, who himself drew out the latter portion of the needles, says that they did not by any means easily come away; and that no blood followed from the puncture. Next in interest to the curious fact of the undoubted extraction of so many needles under the circumstances, is the question as to how they got in the young woman's flesh? Of this she can give no account, having no recollection of having either swallowed them or received them into her body from any external accident; but she says that sometimes she has felt the pricking of needles in her chest, while their progress down the arm was quite perceptible. She states also, that seven years since she recollects that not fewer than fifty similar needles and pieces of needles came from one of her fingers in the course of a fortnight."—*Ibid.*

